# Relationship between Individual External Doses, Ambient Dose Rates and Individuals’ Activity-Patterns in Affected Areas in Fukushima following the Fukushima Daiichi Nuclear Power Plant Accident

**DOI:** 10.1371/journal.pone.0158879

**Published:** 2016-08-05

**Authors:** Wataru Naito, Motoki Uesaka, Chie Yamada, Tadahiro Kurosawa, Tetsuo Yasutaka, Hideki Ishii

**Affiliations:** 1 Research Institute of Science for Safety and Sustainability, National Institute of Advanced Industrial Science and Technology, Tsukuba, Ibaraki, Japan; 2 National Metrology Institute of Japan, National Institute of Advanced Industrial Science and Technology, Tsukuba, Ibaraki, Japan; 3 Institute for Geo-Resources and Environment, National Institute of Advanced Industrial Science and Technology, Tsukuba, Ibaraki, Japan; 4 Fukushima Future Center for Regional Revitalization, Fukushima University, Fukushima, Japan; University of South Carolina, UNITED STATES

## Abstract

The accident at Fukushima Daiichi Nuclear Power Plant on March 11, 2011, released radioactive material into the atmosphere and contaminated the land in Fukushima and several neighboring prefectures. Five years after the nuclear disaster, the radiation levels have greatly decreased due to physical decay, weathering, and decontamination operations in Fukushima. The populations of 12 communities were forced to evacuate after the accident; as of March 2016, the evacuation order has been lifted in only a limited area, and permanent habitation is still prohibited in most of the areas. In order for the government to lift the evacuation order and for individuals to return to their original residential areas, it is important to assess current and future realistic individual external doses. Here, we used personal dosimeters along with the Global Positioning System and Geographic Information System to understand realistic individual external doses and to relate individual external doses, ambient doses, and activity-patterns of individuals in the affected areas in Fukushima. The results showed that the additional individual external doses were well correlated to the additional ambient doses based on the airborne monitoring survey. The results of linear regression analysis suggested that the additional individual external doses were on average about one-fifth that of the additional ambient doses. The reduction factors, which are defined as the ratios of the additional individual external doses to the additional ambient doses, were calculated to be on average 0.14 and 0.32 for time spent at home and outdoors, respectively. Analysis of the contribution of various activity patterns to the total individual external dose demonstrated good agreement with the average fraction of time spent daily in each activity, but the contribution due to being outdoors varied widely. These results are a valuable contribution to understanding realistic individual external doses and the corresponding airborne monitoring-based ambient doses and time-activity patterns of individuals. Moreover, the results provide important information for predicting future cumulative doses after the return of residents to evacuation order areas in Fukushima.

## Introduction

The accident at the Tokyo Electric Power Company (TEPCO) Fukushima Dai-ichi Nuclear Power Plant (F1NPP) in March 2011 discharged radionuclides into the environment and contaminated large areas of land in Japan. Five years after the F1NPP accident, the radiation levels have greatly decreased due to physical decay, weathering, and decontamination operations in Fukushima. Of the 12 municipalities (approximately 146,000 residents) in which the entire or part of the population was forced to evacuate after the F1NPP accident [1], as of March 2016 the evacuation order has been lifted on only three municipalities (i.e., Tamura City, Kawauchi Village, and Naraha Town) and residents are not permitted to live permanently in most of the areas affected by the accident. In addition to the residents forced to leave the evacuation zone, several tens of thousands of people from outside the evacuation zone fled their original homes voluntarily because of anxiety over radiation, and, as of March 2016, about 100,000 people, including former residents of the evacuation zone, continue to live away from their original homes in Fukushima [2]. In order for the government to lift the evacuation order, allowing individuals to return to their original residential areas, it is important to understand the current and future potential individual external doses that these individuals could be exposed to.

The government has designed the decontamination work and the criteria for lifting the evacuation orders on the basis of additional individual external dose estimates using ambient dose levels. The additional individual external dose is calculated by assuming that the background radiation, i.e., the national average exposure due to natural sources of radiation, is 0.04μSv h^-1^, that individuals spend 16 hours indoors and 8 hours outdoors each day, and that the shielding effect of a wooden house is 0.4 times [3]. To express it simply, the additional individual external dose is calculated by the cumulative ambient dose multiplied by a reduction factor of 0.6. Due to these assumptions, the actual individual external dose received is generally considered to be less than the estimated dose. Evidence also indicates that the majority of the measured dose from external radiation is below the estimated dose calculated by the government-proposed simple model [4] [5] [6]. In the rehabilitation stage, it is important to accurately understand or estimate realistic individual external doses so that individuals can make informed decisions based on their radiological protection to return to restricted areas. The government has recently stressed the importance of considering individual external dose data collected from personal dosimeters. For example, in November 2013, the Nuclear Regulation Authority recommended that people wear personal dosimeters once they return to their original home in order to safeguard against radiation exposure [7]. In July 2014, the Ministry of the Environment proposed a new policy that will determine decontamination needs by using radiation exposure data collected using personal dosimeters [8].

Individual external dose monitoring has been conducted in several municipalities in Fukushima [4, 9] and provides realistic external dose values for individuals. Most of the monitoring programs in Fukushima use glass badge dosimeters to obtain the individual external dose. Although glass badge dosimeters are useful in large-scale personal monitoring and can obtain large amounts of data safely and quickly, they clearly cannot identify when and where significant external dose occurs. In order to take appropriate countermeasures against unacceptable doses from external irradiation and to develop an individual external dose model applicable to the affected Fukushima areas, it is important to identify when, where, and how much external exposure occurs, and to quantitatively relate individual external dose and ambient dose rates to different behavior patterns of individuals living in Japanese-style homes. Accurate information on individual external doses is needed by the government policymakers, by people providing health care and radiation dose mitigation advice, and especially by affected citizens.

Several studies have attempted to use personal dosimeters in conjunction with personal diaries to understand realistic individual external dose levels related to time-activity patterns in Fukushima [6, 10, 11]. Takahara et al. [10] measured individual external doses and ambient dose rates and collected the corresponding behavioral patterns of the study participants selected from workers in Fukushima to determine statistical features and causes of variation between individual doses. A group of students at Fukushima High School conducted a study to compare the external radiation exposure levels experienced by students and teachers in Japan, Belarus, France, and Poland, and found that external radiation doses in Fukushima were comparable to those in other parts of Japan and in Europe [11]. Our previous study [6] used a semiconductor silicon personal dosimeter called “D-shuttle” (Chiyoda Technol Inc., Tokyo and AIST, Tsukuba, Japan) together with a global-positioning system (GPS) and Geographical Information System (GIS) device to compare individual external doses and air-borne monitoring-based ambient dose rates in Fukushima. Although these studies contributed valuable information towards understanding realistic individual external dose levels and their variabilities in relation to time-activity patterns in Fukushima, little attention has been given to obtaining radiation exposure information applicable to the prediction of future individual external doses of people with different time-activity patterns.

In the present study, we used “D-Shuttle” personal dosimeters to understand realistic individual external doses and to assess the relationship between individual external dose, ambient dose rates, and activity-patterns of individuals living in the affected areas of Fukushima prefecture. Moreover, we attempted to determine the ranges of parameters applicable to the estimation of future cumulative external doses of individuals by considering time-activity patterns.

## Materials and Methods

### Study Participants and Area

**Fig 1:**
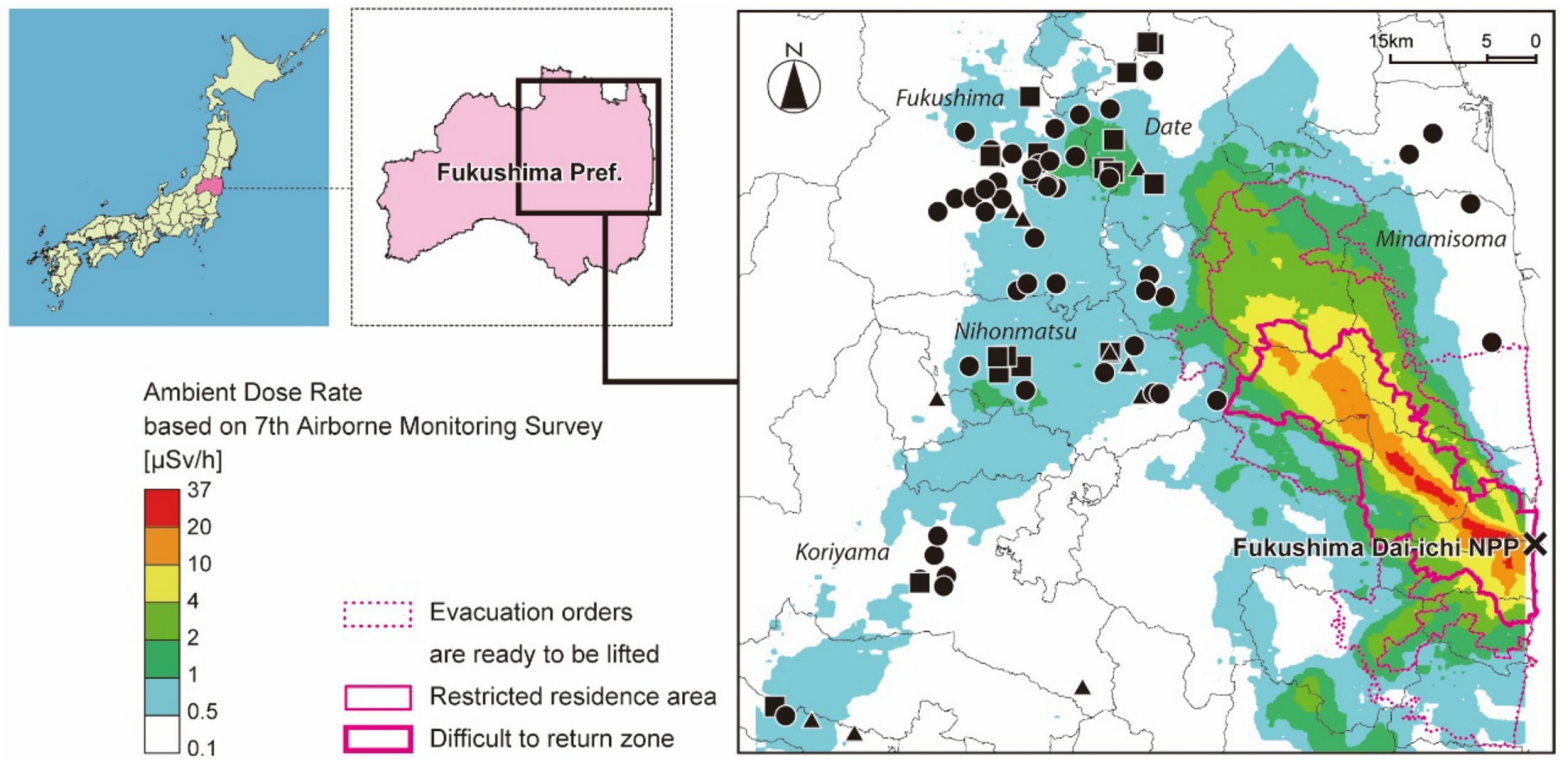
Study area and locations of the homes of study participants. In the figure, circles, triangles, and rectangles represent the homes of subjects who participated in the individual external dose measurements once, twice, and three or more times, respectively. Ambient dose rates adjusted to as of September 28, 2013 are based on the 7th airborne monitoring survey conducted between August 27 and September 28, 2013. Maps created using ArcGIS 10.2 based on Digital Maps (Basic Geospatial Information) provided by Geospatial Information Authority of Japan.

Eighty-seven residents of Fukushima Prefecture participated in our study. The locations of the homes are shown in Fig 1. The study was conducted over approximately 3–14-day periods between September 2013 and March 2015. On measurement days, the airborne-monitoring-based ambient dose rates at the homes of the participants ranged from 0.09–1.28 μSv h^-1^, based on the 7th airborne monitoring survey conducted on between August 27 and September 28 [12].

### Determinations of Individual External Doses, Ambient Doses, and Time-Activity Patterns

**Fig 2:**
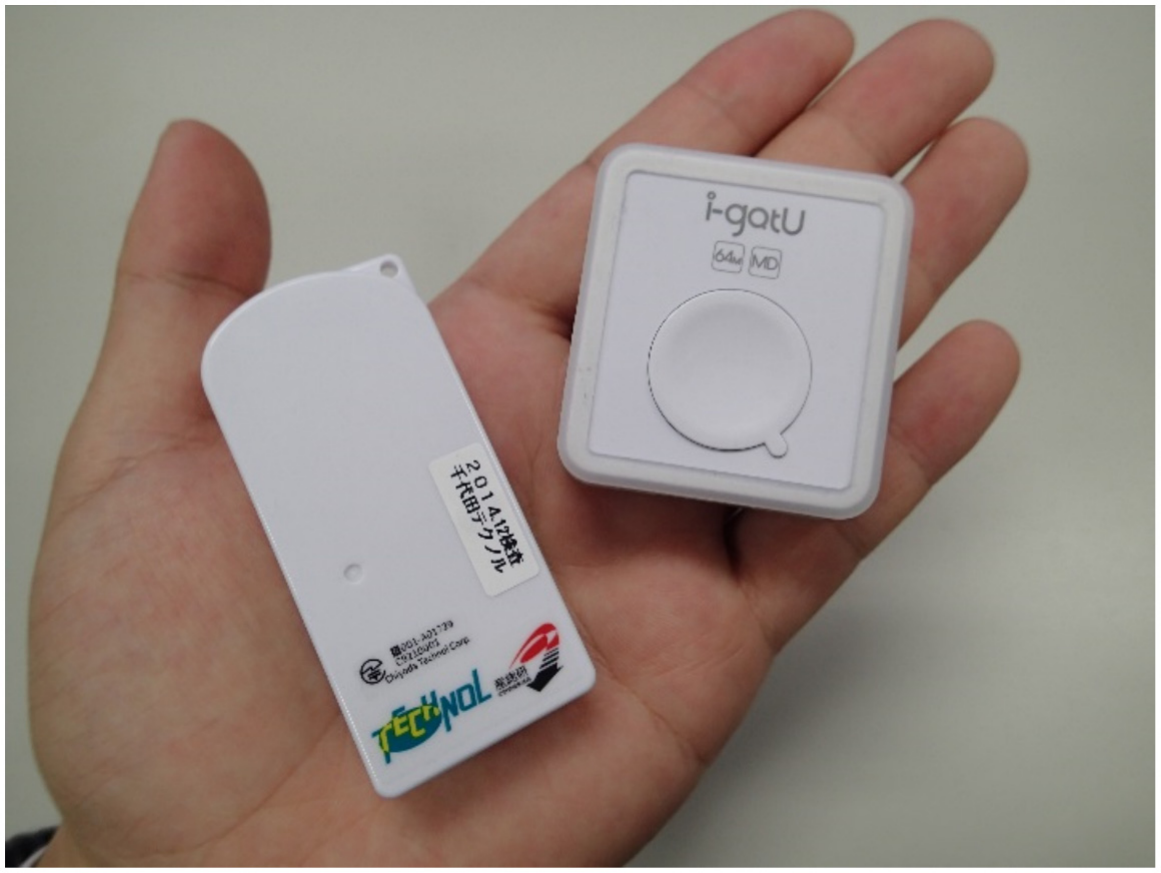
The “D-shuttle” (left) personal dosimeter and “i-gotU” (right) GPS receiver used in this study.

Personal dosimeters incorporating GPS receivers with time-activity diaries and a GIS were used to determine when, where, and how much external exposure occurred. The D-Shuttle was used to determine the hourly and total external dose (Fig 2). The D-Shuttle consists of a silicon semiconductor and can measure total dose ranges of 0.1 μSv to 99.9999 mSv. The sensitivity of the D-Shuttle was calibrated with a ^137^Cs photon source at the Oharai Research Center, Chiyoda Technol Corporation. The values measured by the D-Shuttle are expressed as personal dose equivalent (Hp (10)). Since Hp (10) values measured under the conditions of the affected areas in Fukushima are known to be comparable with the effective dose of isotropic (ISO) or rotational (ROT) irradiation geometries [13, 14], we regarded the individual dose measured by D-shuttle to be a realistic indicator of the effective dose from external radiation exposure. Several municipalities in the special decontamination areas in Fukushima provide D-Shuttle to their residents to measure and understand individual external dose for their own use. Moreover, D-Shuttle is recognized as a good communication tool for understanding individual external doses in affected areas [15].

The i-gotU (GT-600, MobileAction Technology Inc., Taiwan) is a commercial GPS receiver with a data logger (Fig 2) and was used to determine the location of study volunteers at short time intervals. The i-gotU uses the SiRF Star III low power chipset with WASS and EGNOS support. In this study, the i-gotUs were set to record latitude and longitude every 5 seconds. In addition to GPS, self-reported weekly time-activity and location diary data were used to fill any gaps in the GPS data, and to determine indoor and outdoor positions. The GPS and time-activity diary data were used to determine the location and activity of the subjects.

The 7th airborne monitoring survey was used to determine the ambient dose [12]. In relating the individual external dose measured by D-Shuttle with the ambient dose determined based on the 7th airborne monitoring survey, the values of the ambient dose were adjusted for the study periods by taking into account physical decay. Both the individual external dose measured by D-Shuttle and the ambient dose determined based on the 7th airborne monitoring survey include doses resulting from artificial radionuclides (i.e., ^134^Cs and ^137^Cs) and from natural radionuclides. In the analyses of the relationship between the individual external dose and ambient dose, the additional doses resulting from artificial radionuclides were used. Estimation of the ambient dose for the individual external dose measurement was adjusted for the study period, and the physical decay of only artificial radionuclides was taken into account. To calculate additional individual external dose and additional ambient dose, 0.54 mSv/year (i.e., 0.06 μSv h^-1^), set values by Chiyoda Technol Corp. were subtracted from measured value by D-shuttle, and 0.04 μSv h^-1^, set values by the government were subtracted from ambient dose rate determined based on the 7th airborne monitoring survey.

Individual external dose data measured by the D-Shuttle, GPS receiver data with time-activity diaries, and ambient dose rate data were collated into a database by matching the associated timestamps from each device, thereby integrating the data into a common array using ESRI ArcGIS 10.2. The collated data were anonymized then available for post processing and analysis.

### Ethics

This study was approved by the Committee for Ergonomic Experiments at the National Institute of Advanced Industrial Science and Technology (AIST). Written informed consent was obtained from all subjects prior to conducting the study.

## Results and Discussion

### Characteristics of study participants and exposure durations

Eighty-seven residents participated in our study between September 2013 and March 2015; 30 individuals participated multiple times, providing 152 total participants. The data from individuals living in less affected areas and away from Fukushima at the time of the study were eliminated, leaving the data from 142 participants for analysis. Details of study participants and exposure durations are summarized in S1 Table. The participants consisted of 70 full-time farmers, 8 part-time farmers, 35 office workers, 6 housewives, 6 self-employed workers and 17 individuals with an unspecified occupation. According to the diary of each participant, during a given study period, on average the participants spent approximately 57% (SD = 13%) of the time at home, 14% (SD = 11%) of the time indoors other than their home, 16% (SD = 13%) of the time outdoors, 6% (SD = 6%) of the time in a transport vehicle, and 13% (SD = 7%) of the time in an unspecified activity. Our results indicate that people spent a substantial portion of the time at home and this agrees well with time-activity patterns in Fukushima reported elsewhere [10, 16]. In these previous studies, some participants spent more than 50% of the time at home. For outdoor workers, the time spent outdoors was reported to be up to about 40% [10]. In our study, some famers spent approximately 50% of the day outdoors.

### Characteristics of the Measured Individual Doses

**Fig 3:**
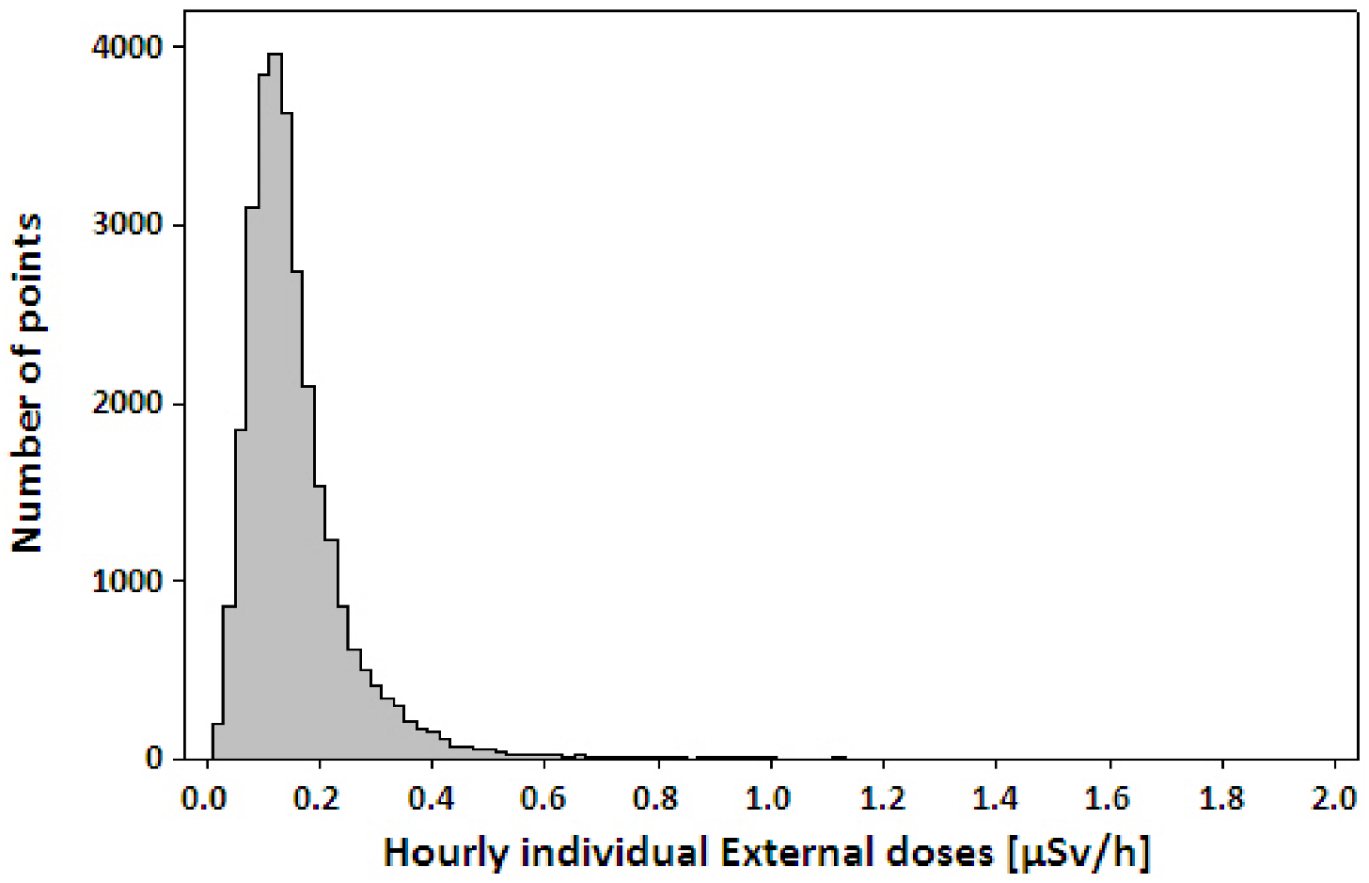
Distribution of hourly individual doses obtained by D-shuttle from study participants.

The dosimeters of the 142 participants provided 29,550 exposure data points, expressed as μSv h^-1^, that were used for analysis (Fig 3, S2 Table). The median and mean of the individual external doses measured by D-shuttle were 0.13 (min:max 0.01:1.86) and 0.16 (Geometric Mean (GM): 0.14) μSv/h.

On average, participants spent 208 (SD = 83) hours carrying the D-shuttle and GPS for data collection. Table 1 lists summary statistics for individual external doses obtained using D-shuttle. The distributions of individual external doses for each person were generally lognormally distributed. More than 6-fold differences were observed in the mean and GM of the individual external doses of participants. The variation in the 95%:5% ratio, expressed by the ratio between the 95th percentiles to the 5th percentile, suggests a large range in inter- and intra-individual activity. The individual external dose measurements obtained using D-shuttle by the Fukushima High School students [11] demonstrated that median hourly individual external doses ranged between 0.07–0.10 μSv/h and this range falls within our results. Of the participants in our study, the person with the highest individual external dose (mean = 0.47 μSv/h) worked in a forest and his house was located in a relatively highly affected area. Other individuals with higher than average individual external doses included a retired person living in a relatively higher ambient dose area, and a farmer working at a non-remediated orchard.

**Table 1:** Summary statistics for individual doses obtained using D-shuttle.

Variable	Median (min—max)	Mean (SD)	GM (GSD)	95th%-ile
Individual dose, n = 142				
Mean for each person	0.14 (0.07–0.47)	0.16 (0.06)	0.15 (1.40)	0.26
SD for each person	0.07 (0.03–0.37)	0.08 (0.06)	0.07(1.69)	0.20
GM for each person	0.13 (0.06–0.39)	0.14 (0.05)	0.13 (1.37)	0.22
GSD for each person	1.59 (1.34–2.80)	1.63 (0.23)	1.62 (1.14)	2.04
95th%-ile for each person	0.26 (0.12–1.22)	0.31 (0.19)	0.28 (1.58)	0.71
95%:5% ratio for each person	4.28 (2.25–18.00)	5.07 (2.73)	4.59 (1.52)	10.32

### Relationship between individual external dose and ambient dose rate

**Fig 4:**
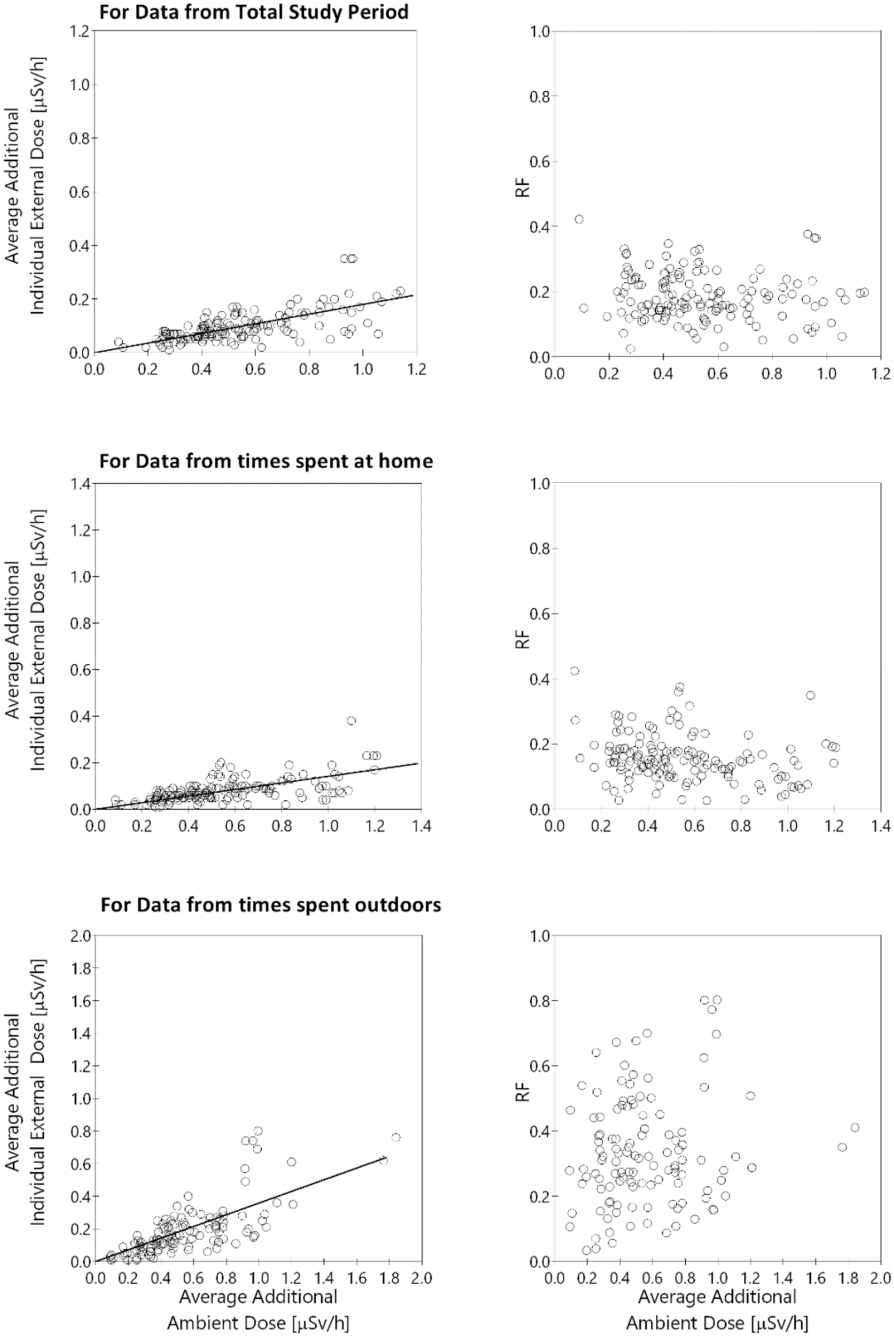
Relationship between average additional individual external dose obtained using D-shuttle and average additional ambient dose estimated based on the 7th air-borne monitoring survey (left) and the relationship between RF (the ratios of individual external doses to ambient doses) and average additional ambient dose (right). The first, second and third rows represent the results for data from the total study period, while at home, and while outdoors, respectively.

The relationship between the average additional external individual dose obtained using D-shuttle and the average additional ambient dose estimated based on the air-borne monitoring survey, and the relationship between the reduction factor (RF) and the average additional ambient dose, are presented in Fig 4. The RF is defined as the ratio of the additional individual external dose to the additional ambient dose. The raw data used to analyze these relationships are provided in S3 Table. Individual external dose data, making it possible to locate position and relate the ambient dose data, were used in this analysis. We observed significant positive correlation between the average additional ambient dose and the additional individual external dose for all cases (p < 0.0001). The regression line showed that the average additional individual external doses were approximately 18%, 14% and 36% of the average additional ambient doses during all study periods, while at home, and while outdoors, respectively. These results indicate that the average additional individual external doses were significantly lower than the average additional ambient doses estimated based on the air-borne monitoring survey. The exposure while at home was estimated to be the lowest mostly due to the building shielding effect. We observed no correlation between the average additional ambient dose and the reduction factor for all study periods (r = -0.11, p = 0.19) and time spent outdoors (r = 0.11, p = 0.22), and a small negative correlation for time spent at home (r = -0.26, p = 0.002). The mean values of the RFs were 0.18 (min-max: 0.02–0.42) for all study periods, 0.16 (min-max: 0.03–0.42) for time spent at home, and 0.32 (min-max: 0.01–0.80) for time spent outdoors. Previous studies also demonstrated that the levels of individual external doses directly measured using a personal dosimeter were substantially lower than levels estimated using the government-proposed equation [5, 6]. The RFs reported in these studies were on average about 0.3 and this is larger than the RFs obtained from the current study. The reasons for this discrepancy include the values of background doses used to estimate additional dose, consideration of decay in the ambient dose determination, and differences in number and type of participants.

Individual external dose, expressed as Hp (10) values, measured in affected areas in Fukushima have previously been reported to be comparable with the ISO or ROT irradiation geometries [13, 14]. Earlier published evidence showed that the values of Hp (10) were about 0.7 times that of the ambient dose rate, expressed as H*(10), if the measurements were conducted in outdoor field conditions in affected areas in Fukushima [17]. In the current study, most of the RFs were derived from outdoor data in which the mean value was 0.32 (min-max: 0.01–0.80), which is much lower than the reported value of 0.7. There can be several reasons for such discrepancies and variabilities among the RFs. The airborne monitoring-based ambient dose rates, which used in the current study, are considered as an aerial average, and not the ambient dose rates based on the point locations of individuals. The airborne monitoring data were obtained using high-sensitivity radiation detectors installed on helicopters: second-by-second measurements are taken of gamma rays emitted from radioactive substances deposited in circles of a diameter approximately twice the flight altitude (target altitude: 150 to 300 m) and centered around ground locations directly below the flight trajectory [18]. If a helicopter flies at an altitude of approximately 300 m above the ground, the system measures the average value of the radiation in a circle in 300 m radius on the ground [18]. A dedicated software program was used to determine the hourly ambient dose rate 1 m above the ground surface at each location based on the value of gamma rays measured in the air and the reading from the survey meter on the ground [18]. Therefore, the airborne monitoring-based ambient dose rates, therefore, may be over or under estimations of the actual ambient dose rates measured on the ground near the locations of individuals. The reasons for very low RFs obtained in this stu294dy can be due to several factors such as decontamination activity, and the behavior and locations of individuals. If an individual stays or works in a decontaminated area and the airborne monitoring system does not detect the decrease in radiation level of the decontamination area, the values for individual external dose can be much lower than the airborne monitoring-based ambient dose rates. In addition, an individual working for a long period of time on tarmacked surfaces or in farm trucks could be exposed to a low individual external doses. Quantifications of the effects of these factors on RFs remain a future challenges.

People are not typically standing motionless outdoors, but rather spend time both outdoors and indoors where there is some sort of radiation shielding. The mean RF for time spent at home obtained in this study was 0.16 (min-max: 0.03–0.42). The median reduction factor with an interquartile range for wooden houses, which is the ratio of indoor ambient dose rate to outdoor ambient dose rate, has been reported to be 0.43 (0.34–0.53) [19]. Since the methods for calculating home RFs in our study and the reported reduction factors are different, it is not possible to directly compare our home RFs and the reported reduction factors. The reported reduction factors are defined as the ratio of indoor ambient dose rate to outdoor ambient dose rate, while the home RFs in our study are defined as the ratio of additional individual dose rate to airborne-based additional ambient dose rate. Since decontamination works has been conducted in the most of houses in the affected areas in Fukushima, the outdoor ambient dose rate measured at decontaminated points around a house are considered to be lower than the airborne-based ambient dose rate. Moreover, the values of Hp (10) were about 0.7 times that of the ambient dose rate [17]. Taking these relationships and variability of RFs into account, our estimated home RFs are comparable to those reported in previous studies [10, 19] and can be used to estimate individual external dose during time spent at home based on the airborne-based ambient dose.

In the current study, we used publicly available ambient dose rate data estimated from the airborne monitoring survey provided by the Japanese government. More accurate predictions of individual external doses could be possible if ambient dose rates and reduction factors corresponding to an individual’s activity locations were prepared, but this would be impossible to perform for a large number of individuals. The current study relates the individual external doses to the publicly available ambient dose rates and with the activity-patterns of the individuals. The results provide, from a practical viewpoint, valuable information for understanding and estimating realistic individual external doses in the affected areas in Fukushima, especially for evacuees who want to know their individual external doses after returning to their original homes.

### Contribution of various activity patterns to the total individual dose

**Fig 5:**
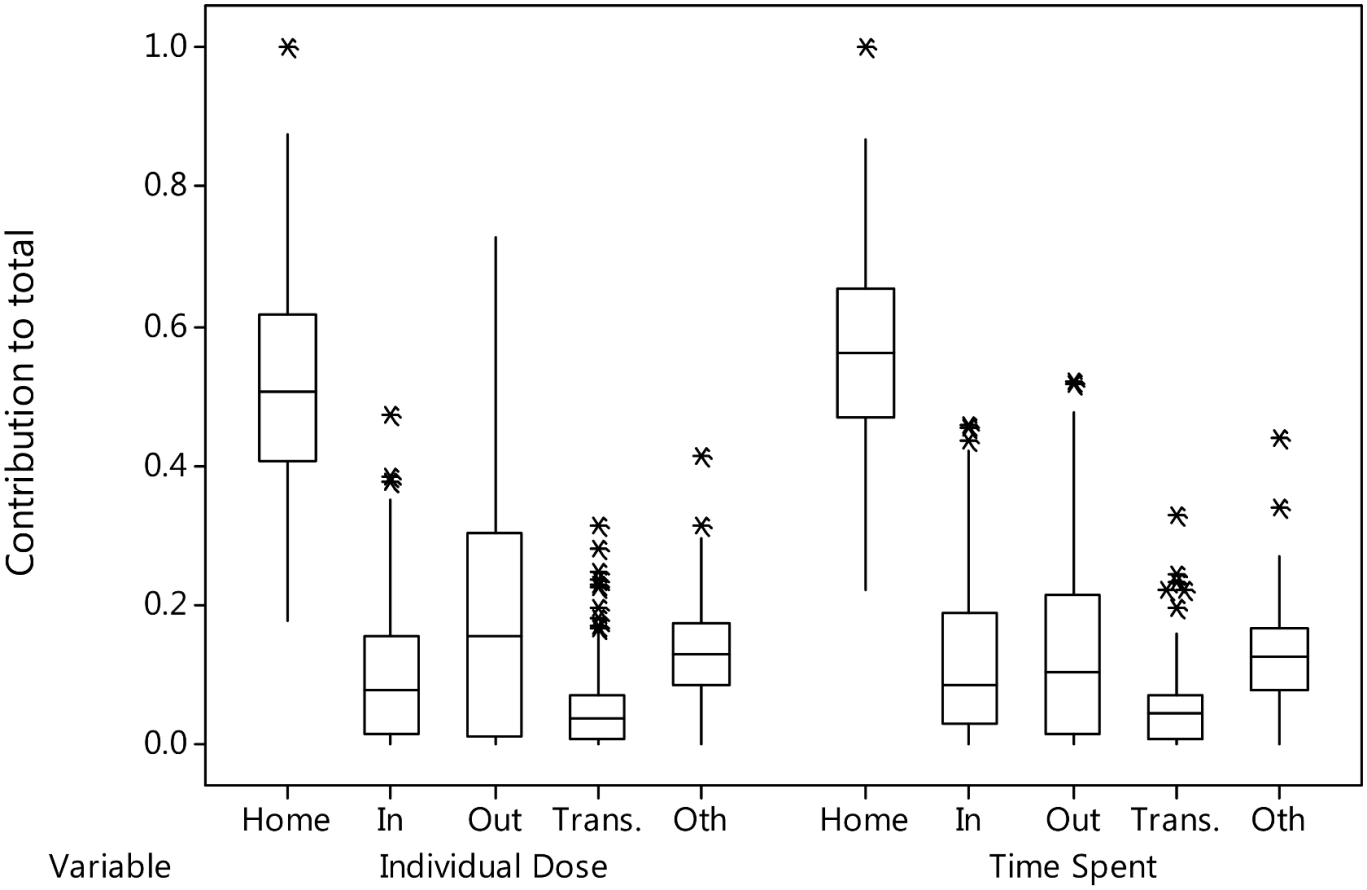
Distribution of contributions of various activities to the total individual dose and the fraction of time spent in each activity. Home: Time spent at home; In: Time spent inside buildings other than the home; Out: Time spent outdoors; Trans: Time spent in transportation; Oth: Unknown. In the box-and-whisker plot, the bottom and top of the box are the first and third quartiles, and the band inside the box is the median. Outliers are identified by asterisks.

As seen in Fig 5, the average contribution of each activity to the total individual external dose agreed well with the average time fraction of the day spent in each activity as determined by GPS and the personal diary. As expected, time spent at home provided a large contribution to the total individual external dose, accounting on average for approximately 50% of the total individual external dose. The contribution of being outdoors varied widely. Time spent outdoors contributed on average approximately 20% of the total individual external dose, but for some farmers it contributed more than 70% of the total exposure due to the farmer spending long periods outdoors in fields with relatively high ambient doses. These results suggest that quantification of individual external doses obtained while at home is essential for examining dose reduction measures for individuals, and that detailed monitoring data are required to understand the contribution of outdoor activities to the total individual external dose.

## Conclusions

We used D-shuttle personal dosimeters along with GPS and GIS to relate individual external doses, ambient doses and activity-patterns of individuals in affected areas in Fukushima. The measured individual external doses were well correlated to the ambient doses based on the 7th airborne monitoring survey and the results of linear regression analysis suggested that the additional individual external doses were on average about one-fifth and one-third of the additional ambient doses and the individual external dose estimated by the government proposed formula, respectively. Analysis of the contribution of various activity patterns to the total individual external dose demonstrated good agreement with the average time fraction spent on each activity each day, but the contribution due to being outdoors varied widely. The current results are a valuable contribution to understanding realistic individual external doses, and the corresponding time-activity patterns and airborne monitoring air dose rate. Moreover, the results provide important information for predicting future cumulative external doses following the return of residents to their homes in the evacuation order areas in Fukushima.

## Supporting Information

S1 Table Details of study participants and exposure durations. (DOCX)

S2 Table Hourly individual external dose data measured by D-shuttle for the 142 participants. (XLSX)

S3 Table Data on individual external dose and airborne-based ambient dose for analyzing the relationship. Individual external dose data, making it possible to locate position and relate the ambient dose data, were provided.
(XLSX)
