# Supplementary material for: Relationship between Individual External Doses, Ambient Dose Rates and Individuals’ Activity-Patterns in Affected Areas in Fukushima following the Fukushima Daiichi Nuclear Power Plant Accident
Source: PLoS One. 2016 Aug 5;11(8):e0158879. doi: 10.1371/journal.pone.0158879 (PMC4975410; doi:10.1371/journal.pone.0158879)
Supplement: S1 Table — (DOCX) [file pone.0158879.s001.docx]

S1Table

| Subject  No. | Total  Hours | Hours Individual  Dose Data Obtained | Hours the location of  the subject identified | Hours for Analysis | Occupation |
| --- | --- | --- | --- | --- | --- |
| 1 | 181 | 181 | 176 | 176 | Office worker |
| 2 | 186 | 186 | 174 | 174 | Office worker |
| 3 | 186 | 186 | 182 | 182 | Office worker |
| 4 | 186 | 186 | 185 | 185 | Office worker |
| 5 | 85 | 85 | 78 | 78 | Office worker |
| 6 | 186 | 186 | 186 | 186 | Office worker |
| 7 | 210 | - | - | - | Office worker |
| 8 | 180 | 180 | 177 | 177 | Office worker |
| 9 | 90 | 89 | 84 | 83 | Office worker |
| 10 | 229 | 229 | 218 | 218 | Full-time farmer |
| 11 | 234 | 234 | 234 | 234 | Full-time farmer |
| 12 | 239 | - | - | - | Part-time farmer |
| 13 | 234 | - | - | - | Full-time farmer |
| 14 | 210 | 210 | 205 | 205 | Full-time farmer |
| 15 | 301 | 301 | 271 | 271 | Other |
| 16 | 186 | 186 | 178 | 178 | Full-time farmer |
| 17 | 275 | 275 | 263 | 263 | Full-time farmer |
| 18 | 184 | 184 | 183 | 183 | Full-time farmer |
| 19 | 138 | 114 | 111 | 111 | Full-time farmer |
| 20 | 186 | 162 | 160 | 160 | Other |
| 21 | 108 | 108 | 108 | 108 | Full-time farmer |
| 22 | 144 | 144 | 144 | 144 | Full-time farmer |
| 23 | 168 | 168 | 140 | 140 | Office worker |
| 24 | 162 | 162 | 162 | 162 | Other |
| 25 | 282 | 258 | 258 | 258 | Office worker |
| 26 | 378 | 378 | 378 | 378 | Office worker |
| 27 | 162 | 162 | 157 | 157 | Part-time farmer |
| 28 | 210 | 210 | 203 | 203 | Office worker |
| 29 | 210 | 186 | 178 | 178 | Other |
| 30 | 210 | 186 | 181 | 181 | Other |
| 31 | 282 | 281 | 281 | 280 | Self-employed business |
| 32 | 306 | 305 | 291 | 290 | Office worker |
| 33 | 474 | - | - | - | Other |
| 34 | 205 | 205 | 204 | 204 | Office worker |
| 35 | 300 | 300 | 215 | 215 | Other |
| 36 | 570 | 402 | 371 | 371 | Other |
| 37 | 378 | 378 | 362 | 362 | Other |
| 38 | 558 | - | - | - | Full-time farmer |
| 39 | 186 | - | - | - | Self-employed business |
| 40 | 402 | - | - | - | Full-time farmer |
| 41 | 378 | 378 | 345 | 345 | Other |
| 42 | 306 | 306 | 223 | 223 | Other |
| 43 | 378 | - | - | - | Full-time farmer |
| 44 | 376 | 376 | 349 | 349 | Other |
| 45 | 354 | - | - | - | Housewife |
| 46 | 144 | 144 | 141 | 141 | Office worker |
| 47 | 72 | 72 | 72 | 72 | Office worker |
| 48 | 208 | 208 | 200 | 200 | Office worker |
| 49 | 248 | 248 | 248 | 248 | Office worker |
| 50 | 288 | - | - | - | Office worker |
| 51 | 186 | 186 | 181 | 181 | Self-employed business |
| 52 | 234 | 234 | 221 | 221 | Full-time farmer |
| 53 | 162 | 162 | 153 | 153 | Part-time farmer |
| 54 | 356 | 356 | 345 | 345 | Other |
| 55 | 186 | 186 | 175 | 175 | Full-time farmer |
| 56 | 378 | 378 | 363 | 363 | Full-time farmer |
| 57 | 192 | 192 | 176 | 176 | Full-time farmer |
| 58 | 139 | 139 | 138 | 138 | Full-time farmer |
| 59 | 142 | 142 | 139 | 139 | Office worker |
| 60 | 124 | 124 | 124 | 124 | Office worker |
| 61 | 133 | 133 | 130 | 130 | Office worker |
| 62 | 165 | 165 | 150 | 150 | Full-time farmer |
| 63 | 186 | 162 | 159 | 159 | Housewife |
| 64 | 126 | 126 | 126 | 126 | Office worker |
| 65 | 156 | 156 | 144 | 144 | Full-time farmer |
| 66 | 159 | 111 | 110 | 110 | Full-time farmer |
| 67 | 333 | 333 | 315 | 315 | Office worker |
| 68 | 186 | 186 | 178 | 178 | Full-time farmer |
| 69 | 114 | 114 | 114 | 114 | Full-time farmer |
| 70 | 450 | - | - | - | Office worker |
| 71 | 170 | 170 | 168 | 168 | Office worker |
| 72 | 318 | 318 | 289 | 289 | Office worker |
| 73 | 159 | 159 | 151 | 151 | Office worker |
| 74 | 208 | 208 | 208 | 208 | Office worker |
| 75 | 210 | 210 | 207 | 207 | Part-time farmer |
| 76 | 257 | 209 | 200 | 200 | Housewife |
| 77 | 186 | 186 | 186 | 186 | Other |
| 78 | 258 | 210 | 210 | 210 | Self-employed business |
| 79 | 186 | 186 | 186 | 186 | Full-time farmer |
| 80 | 162 | 162 | 162 | 162 | Housewife |
| 81 | 162 | 162 | 162 | 162 | Full-time farmer |
| 82 | 155 | 155 | 155 | 155 | Office worker |
| 83 | 174 | 174 | 168 | 168 | Full-time farmer |
| 84 | 162 | 162 | 162 | 162 | Office worker |
| 85 | 162 | 162 | 161 | 161 | Full-time farmer |
| 86 | 180 | 180 | 180 | 180 | Full-time farmer |
| 87 | 147 | 147 | 146 | 146 | Full-time farmer |
| 88 | 192 | 192 | 179 | 179 | Full-time farmer |
| 89 | 195 | 171 | 169 | 169 | Full-time farmer |
| 90 | 156 | 156 | 153 | 153 | Full-time farmer |
| 91 | 162 | 162 | 162 | 162 | Full-time farmer |
| 92 | 186 | 186 | 180 | 180 | Part-time farmer |
| 93 | 42 | - | - | - | Other |
| 94 | 199 | 199 | 189 | 189 | Full-time farmer |
| 95 | 162 | 162 | 149 | 149 | Full-time farmer |
| 96 | 186 | 186 | 182 | 182 | Full-time farmer |
| 97 | 210 | 210 | 179 | 179 | Full-time farmer |
| 98 | 175 | 175 | 170 | 170 | Full-time farmer |
| 99 | 186 | 186 | 184 | 184 | Part-time farmer |
| 100 | 210 | 210 | 202 | 202 | Full-time farmer |
| 101 | 210 | 210 | 210 | 210 | Part-time farmer |
| 102 | 162 | 162 | 160 | 160 | Office worker |
| 103 | 162 | 162 | 162 | 162 | Office worker |
| 104 | 162 | 162 | 162 | 162 | Other |
| 105 | 162 | 162 | 162 | 162 | Other |
| 106 | 186 | 186 | 110 | 110 | Full-time farmer |
| 107 | 234 | 234 | 216 | 216 | Full-time farmer |
| 108 | 210 | 210 | 210 | 210 | Full-time farmer |
| 109 | 162 | 162 | 161 | 161 | Full-time farmer |
| 110 | 162 | 162 | 162 | 162 | Office worker |
| 111 | 234 | 162 | 160 | 160 | Office worker |
| 112 | 162 | 162 | 161 | 161 | Full-time farmer |
| 113 | 162 | 162 | 162 | 162 | Full-time farmer |
| 114 | 282 | 282 | 275 | 275 | Full-time farmer |
| 115 | 186 | 186 | 185 | 185 | Full-time farmer |
| 116 | 378 | 329 | 315 | 314 | Housewife |
| 117 | 186 | 186 | 186 | 186 | Full-time farmer |
| 118 | 199 | 199 | 152 | 152 | Full-time farmer |
| 119 | 162 | 162 | 161 | 161 | Full-time farmer |
| 120 | 162 | 162 | 127 | 127 | Full-time farmer |
| 121 | 197 | 197 | 197 | 197 | Full-time farmer |
| 122 | 210 | 210 | 207 | 207 | Part-time farmer |
| 123 | 159 | 159 | 159 | 159 | Full-time farmer |
| 124 | 210 | 210 | 206 | 206 | Full-time farmer |
| 125 | 162 | 162 | 162 | 162 | Housewife |
| 126 | 162 | 162 | 162 | 162 | Housewife |
| 127 | 186 | 186 | 184 | 184 | Full-time farmer |
| 128 | 210 | 210 | 197 | 197 | Full-time farmer |
| 129 | 191 | 191 | 190 | 190 | Full-time farmer |
| 130 | 162 | 162 | 162 | 162 | Self-employed business |
| 131 | 234 | 234 | 227 | 227 | Self-employed business |
| 132 | 186 | 186 | 186 | 186 | Full-time farmer |
| 133 | 372 | 372 | 365 | 365 | Full-time farmer |
| 134 | 207 | 207 | 177 | 177 | Full-time farmer |
| 135 | 378 | 378 | 351 | 351 | Full-time farmer |
| 136 | 378 | 378 | 362 | 362 | Self-employed business |
| 137 | 373 | - | - | - | Part-time farmer |
| 138 | 395 | 365 | 329 | 323 | Full-time farmer |
| 139 | 378 | 378 | 359 | 359 | Full-time farmer |
| 140 | 378 | 378 | 365 | 365 | Full-time farmer |
| 141 | 757 | 733 | 721 | 721 | Other |
| 142 | 186 | 186 | 172 | 172 | Full-time farmer |
| 143 | 186 | 186 | 176 | 176 | Full-time farmer |
| 144 | 162 | 138 | 138 | 138 | Full-time farmer |
| 145 | 186 | 186 | 172 | 172 | Full-time farmer |
| 146 | 186 | 186 | 186 | 186 | Full-time farmer |
| 147 | 162 | 162 | 162 | 162 | Full-time farmer |
| 148 | 180 | 179 | 178 | 177 | Full-time farmer |
| 149 | 159 | 158 | 134 | 134 | Full-time farmer |
| 150 | 252 | 252 | 221 | 221 | Other |
| 151 | 186 | 185 | 94 | 94 | Part-time farmer |
| 152 | 162 | 162 | 144 | 144 | Full-time farmer |
| 153 | 426 | 234 | 175 | 175 | Office worker |
| 154 | 426 | 210 | 176 | 176 | Office worker |
| 155 | 186 | 185 | 135 | 135 | Full-time farmer |
| 156 | 375 | - | - | - | Other |
| 157 | 384 | - | - | - | Other |
